# Severe hyponatraemia (P-Na < 116 mmol/l) in the emergency department: a series of 394 cases

**DOI:** 10.1007/s11739-023-03221-y

**Published:** 2023-02-17

**Authors:** Sami Mustajoki

**Affiliations:** Department of Medicine, Tampere University Hospital, P.O. Box 2000, 33521 Tampere, Finland

**Keywords:** Hyponatraemia, Sodium correction, Emergency department, Mortality, Osmotic demyelination syndrome

## Abstract

**Aim:**

To evaluate the significance of severe hyponatraemia presented at the emergency department (ED).

**Methods:**

A retrospective hospital records study of all patients with plasma sodium levels of < 116 mmol/l from 2016 to 2020 in a single tertiary referral centre.

**Results:**

A total of 394 visits of 363 individual severely hyponatraemic patients represented 0.08% of all ED visits. The mean age was 68 years and the male-to-female ratio was 1:1.3. The symptoms and signs were diffuse and varying, while half of the patients had neurologic symptoms. The aetiology of hyponatraemia was often multifactorial. The aetiologies varied by age, and the most common ones were the syndrome of inappropriate antidiuresis (34%), diuretic use (27%), alcohol-related (19%) and dehydration (19%). The mean sodium correction rates were 6.6, 4.9 and 3.8 mmol/l/24 h at 24, 48 and 72 h, respectively. The mean maximum correction rate over any 24-h time interval was 10.2 mmol/l. The vital signs (National Early Warning Score, NEWS) of severely hyponatraemic patients were mostly normal. All-cause mortality was 18% for 1-year follow-up. Malignancies, especially small-cell lung cancer, and end-stage liver disease caused most of the deaths. Osmotic demyelination syndrome (ODS) was diagnosed in five (1.4%) patients.

**Conclusion:**

Patients with severe hyponatraemia in the ED presented with non-specific complaints. The aetiology of hyponatraemia was often multifactorial and varied by age. The need for intensive care was poorly predicted by NEWS. The one-year mortality rate was 18% and the incidence of ODS 1.4% after an episode of severe hyponatraemia.

## Introduction

Hyponatraemia is the most common electrolyte disturbance [1] and it is associated with increased mortality and morbidity [2]. Hyponatraemia is present in 2%–10% of patients in the emergency department (ED) [3–6]. Most hyponatraemias are mild, and extremely low sodium concentrations are infrequent. However, severe hyponatraemia is potentially life-threatening, and it predisposes the patient to osmotic demyelination syndrome (ODS). The importance of a prompt diagnostic workup and management is emphasized in cases of severe hyponatraemia [7].

Previous reports have evaluated the significance of patients with hyponatraemia upon admission, including patients with severe hyponatraemia [2–6, 8]. However, little is known about the aetiology and detailed outcome of severely hyponatraemic patients presenting at the emergency department.

This report describes a series of 394 consecutive patients with severe hyponatraemia (P-Na < 116 mmol/l) in the emergency department of a single tertiary referral university hospital. The aim of this retrospective study was to investigate the epidemiology, aetiology, clinical manifestations, management and outcome of patients with profound hyponatraemia upon admission to the ED.

## Material and methods

A retrospective examination of hospital records was carried out at Tampere University Hospital, Finland. This hospital provides services for 500,000 inhabitants and is a tertiary-level hospital for a total population of 900,000. The hospital’s ED has approximately 90,000 visits annually, mostly comprising adult patients.

The database of the hospital’s laboratory (Fimlab Laboratoriot Oy Ltd) was searched to identify all patients admitted to the emergency department (ED) with plasma sodium (P-Na) concentrations < 116 mmol/l over a five-year period between 1 January 2016 and 31 December 2020. The P-Na results reported by the laboratory were not adjusted for the concentrations of plasma glucose. All ED visits were included in the analysis, and each patient may, thus, have had one or more admissions with severe hyponatraemia. However, when calculating the one-year mortality, only the first visit of each patient was used as a starting point.

One investigator reviewed data from patient charts and made evaluations and analyses. The following data were obtained: age, sex, time of death, cause of death, referring instance, reported symptoms upon admission, systolic blood pressure, heart rate, respiratory rate, body temperature, oedema, medication, prior hospitalization or change in medication within 30 days, initial therapy and all hyponatraemia-targeted treatments during hospitalization, length of hospitalization at the university hospital, first and second wards during hospitalization, and location after university hospital discharge. Medications were categorized as diuretics, antiepileptics, antidepressants, antipsychotics, chemotherapy, proton pump inhibitors, non-steroidal anti-inflammatory drugs, as well as other medications, including corticosteroids and opiates.

The diagnosis of osmotic demyelination syndrome (ODS) was made if typical findings in magnetic resonance imaging (MRI) scan were documented.

If the National Early Warning Score (NEWS) [9] was not recorded, the investigator reconstructed the scores using available information. The Glasgow Coma Scale (GCS) [10] was not systematically recorded in patient charts since it is not required data in the NEWS. However, verbal expressions such as ‘alert’ or ‘normal consciousness’ were considered to represent 15 points on the GCS. The assessment of eventual excessive alcohol use was based on the investigator’s judgement based on available records.

The cause of hyponatraemia in each case was independently evaluated by the investigator, regardless of the clinicians’ judgment during the hospitalization. The aetiology of hyponatraemia was based on patient history, medication, clinical findings, especially those indicating evaluation of volume status, and laboratory results. Generally, if a spot urine sodium concentration was at least 30 mmol/l, urine osmolality was at least 200 mosm/kg H_2_O and no apparent other reason for natriuresis was present, syndrome of inappropriate antidiuresis (SIAD) was considered as the aetiology of hyponatraemia. In addition, SIAD was also diagnosed even without a result of a spot urine sodium concentration, if this diagnosis otherwise seemed obvious – for example, in cases of postoperative nausea. Alcohol-related reasons for hyponatraemia were handled as one group, since the low solute intake in beer potomania and SIAD due to alcohol withdrawal syndrome, for example, were often concurrent when hyponatraemia was diagnosed [11].

During the study period, there was no standardized protocol for the management of hyponatraemia in the hospital. Thus, the choice of the management modality was made by a heterogenous group of clinicians including emergency medicine doctors, anaesthesiologists and internists, either specialists or doctors in training.

Sodium analyses were performed either on an arterial sample, using a point-of-care blood gas analyser ABL90Flex (Radiometer Medical), or on venous blood, using ion-selective electrodes on the Roche Diagnostics Cobas c501 before June 2018 and on the Cobas c702 thereafter. All P-Na measurements during the initial 72 h after admission were included in the analysis. For each case, the estimated P-Na at 24, 48 or 72 h was calculated as described earlier by George et al. [12], using the closest sodium values before and after the desired time mark and assuming a linear change between these two data points. Furthermore, the maximal 24-h change in P-Na at any time point during the initial 72 h was calculated using the same interpolation method. Estimated P-Na change was also calculated both 24 h backwards and 24 h forwards from every sodium measurement available.

In addition to P-Na levels, the first result after admission was recorded for the following laboratory variables in blood, plasma or serum: haemoglobin, leukocytes, INR, C-reactive protein, potassium, creatinine, albumin, ionized calcium, alanine aminotransferase, bilirubin, thyroid stimulating hormone, pH, base excess, lactate and cortisol. Urine osmolality and sodium concentration were recorded.

Data was recorded and descriptive statistics (mean, median, range, standard deviation (SD) and inter-quartile range (IQR)) were calculated using Microsoft Excel 365 software. Gender-adjusted odds ratios (OR) for age, confidence intervals (CI) and p-values for aetiologies of hyponatraemia were calculated using RStudio Desktop version 2022.02.2 + 485. Age was considered as continuous variable.

According to Finnish legislation, hospital ethics committee approval is not required for registered studies. The study was duly approved by the hospital’s research director (research diary number R21513).

## Results

During the study period of five years, 240,993 sodium analyses were performed in the emergency department of Tampere University Hospital. The results of 59,726 (24.8%) analyses were below the reference range of 137–144 mmol/l. The database search revealed 413 results (0.17% of all sodium analyses) meeting the inclusion criterion (P-Na < 116 mmol/l). Finally, 394 individual ED visits with severe hyponatraemia were detected, representing 0.08% of all 468,756 ED visit during the study period.

Of the patients included in the study, 336 (93%) had a single ED visit meeting the inclusion criterion, 24 patients (7%) were admitted twice, while three patients (1%) had three or more ED visits during which their P-Na was 115 mmol/l or lower. The mean age of the patients was 68 years, ranging from 17 to 98 years. The male-to-female ratio was 1:1.3, but males predominated the two youngest age quartiles (Table 1).

**Table 1:** Characteristics of patients with severe hyponatraemia (P-Na < 116 mmol/l)

	All patients		Q1	Q2	Q3	Q4
			17–58 yrs	58–68 yrs	68–79 yrs	79–98 yrs
Total visits in ED	394		98	99	99	98
Individual patients	363		87	88	94	94
1 visit	336	(93%)	79	78	89	90
2 visits	24	(7%)	6	9	5	4
3 visits or more	3	(1%)	2	1	0	0
Mean age, years (range)	68	(17–98)	48	63	74	85
Female	222	(56%)	32	43	64	83
Arriving at ED						
Without referral	233	(59%)	64	64	46	59
Referral from outpatient clinic	111	(28%)	24	25	36	26
Primary care ward	23	(6%)	2	3	6	12
Other hospital	18	(5%)	5	4	9	0
Other institution	9	(2%)	3	3	2	1
Medication						
No. of prescribed drugs, mean	5.7		4.0	5.0	6.7	7.0
Any change within 1 month	111	(28%)	16	20	20	38
Any diuretic	164	(42%)	22	35	45	62
HCTZ or indapamide	110	(28%)	15	17	36	42
PPI	106	(27%)	24	28	30	24
Antidepressants	84	(21%)	28	25	12	19
SSRI	60	(15%)	20	16	9	15
Antipsychotics	64	(16%)	26	14	17	7
Antiepileptics	31	(8%)	8	10	8	5
Carbamazepine	2	(1%)	1	1	0	0
Antidiuretic hormone analogue	3	(1%)	0	1	1	1
Alcohol abuse	142	(36%)	65	48	23	6

*ED* emergency department, *HCTZ* hydrochlorothiazide, *PPI* proton pump inhibitor, *SSRI* selective serotonin reuptake inhibitor

The mean number of prescribed drugs was 5.7, with an increasing trend with age (Table 1), while 31 (8%) patients had no previous medications. In 28% of the cases, the medication had been altered within 30 days prior to the ED visit, including the initiation of therapy with an antibiotic in 29 patients, an opiate in 14 patients, a selective serotonin uptake inhibitor (SSRI) in 12 patients and a diuretic in 10 patients. Thirty-five patients (9% of all cases) had been hospitalized for various reasons within 30 days prior to the ED visit.

### Symptoms

The reported symptoms upon admission to the ED are listed in Table 2. More than one symptom was reported by 226 (57%) patients. Symptoms were divided into the solely neurological group and the other symptoms group, which may also include symptoms of neurologic origin, such as impaired cognition, muscle weakness, convulsions or impaired consciousness. Other neurological symptoms included vertigo, as well as aphasia or other symptoms mimicking ischemic stroke. Lethargy was a frequent complaint, representing a condition during which the general performance was deteriorated, usually without any specific symptom. Nausea or vomiting was considered as symptoms of moderate or severe hyponatraemia, respectively. Dyspnoea and oedema were regarded as signs of hypervolaemia. Only one patient with a coincidental finding of severe hyponatraemia was totally asymptomatic.

**Table 2:** Reported symptoms upon admission

Average no. of symptoms (range)	1.8	(0–5)
Neurological symptoms	195	(49%)
Confusion or disorientation	82	(21%)
Muscle weakness or falling	80	(20%)
Convulsion	39	(10%)
Impaired consciousness	20	(5%)
Other neurological	32	(8%)
Lethargy	141	(36%)
Nausea or vomiting	125	(32%)
Pain	59	(15%)
Diarrhoea	38	(10%)
Dyspnoea or oedema	35	(9%)
Other	51	(13%)

### Hospitalization

After the emergency department visit, 384 (97%) patients were admitted to the hospital and 4 patients to a primary care ward, and 4 patients were discharged. Two patients died in the ED. Ninety-seven (25%) patients were admitted to the intensive care unit (ICU), 83 (21%) to a high-dependency unit (HDU), 174 (44%) to internal medicine wards and 30 (8%) to other wards. The HDUs included the internal medicine HDU (67 patients), stroke unit (8 patients), cardiac care unit (7 patients) and pulmonary HDU (1 patient). Altogether, 204 (52%) patients were admitted to the ICU or HDU during hospitalization.

The mean length of stay at the university hospital was 7.7 days (range 0–122 days), while hospitalization exceeded 14 days in 42 (11%) patients. Half of the patients were discharged and returned home, 151 (38%) were transferred to a primary care unit or other hospital, 28 (7%) died during hospitalization, and 17 (4%) were transferred to an institution, including psychiatric wards.

### National early warning score and Glasgow coma scale

A NEWS was available in 240 (61%) cases upon admission, and in the remaining 154, the score was reconstructed retrospectively using information available in the patient records. The score was from 0 to 2 in 256 (65%) cases, from 3 to 5 in 96 (24%) cases, from 6 to 8 in 27 (7%) cases, and 9 or higher in 15 (4%) cases. Ninety-one (36%) of the patients scoring up to 2 points and 87% (13 out of 15) those scoring at least 9 points were admitted to the ICU or an HDU. The average score was 3.5 (median 3.0) in severely hyponatraemic patients admitted to the ICU.

Data for the evaluation of consciousness were available for 372 patients, and 92% of them scored a GCS of 14 or 15. A GCS of 8 or lower was reported in 12 (3%) patients.

### Causes of hyponatraemia

Data for urine sodium, urine osmolality and serum osmolality were available in 340, 328 and 262 cases, respectively. The causes of hyponatraemia, divided into age quartiles, are presented in Table 3. Hyponatraemia was multifactorial in 44% of the patients, and the average number of aetiologies was 1.5.

**Table 3:** Causes of hyponatraemia

					17–58 yrs	58–68 yrs	68–79 yrs	79–98 yrs
					*n* = 98	*n* = 99	*n* = 99	*n* = 98
Average no. of causes (range)			1.5	(0–4)	1.5	1.5	1.5	1.6
Inappropriate antidiuresis (SIAD)*			134	(34%)	16	27	38	53
Pain, nausea, vomiting*			51	(13%)	7	5	19	20
Malignancy			20	(5%)	1	10	4	5
Medication			17	(4%)	3	4	2	8
Pulmonary			12	(3%)	2	3	6	1
Other or unspecified*			48	(12%)	5	9	9	25
Diuretic*			108	(27%)	14	20	31	43
Beer potomania or other alcohol-related*			96	(24%)	38	38	17	3
Polydipsia			73	(19%)	17	15	21	20
Dehydration			73	(19%)	18	24	19	12
Hypervolemia			55	(14%)	20	12	10	13
Cardiac*			24	(6%)	2	3	6	13
Hepatic*			25	(6%)	15	7	3	0
Other or unspecified			6	(2%)	3	2	1	0
Acute kidney injury			7	(2%)	5	2	0	0
Hyperglycaemia			6	(2%)	5	1	0	0
Urine retention			6	(2%)	0	2	2	2
Gastrointestinal			5	(1%)	1	0	3	1
Antidiuretic hormone analogue			3	(1%)	0	1	1	1
Addison’s disease			3	(1%)	2	0	1	0
Other			2	(1%)	1	1	0	0
Not known			4	(1%)	0	1	2	1

*Statistical significance for the influence of age. See text for more information

Syndrome of inappropriate antidiuresis was the most common reason for hyponatraemia, seen in 133 (34%) patients. Pain, nausea or vomiting was considered to be a contributing factor in hyponatraemia in 51 (38% of all causes of SIAD) patients. SIAD due to malignancy was the aetiology in 21 cases, 17 of which were small-cell lung cancer (SCLC) patients. The other malignancies comprised two cases of breast cancer, one lymphoma and one case of large-cell lung cancer. Of note, the number of patients with SCLC was 14, since three of the SCLC patients had two ED visits due to hyponatraemia. The medications contributing to hyponatraemia through the SIAD mechanism were selective serotonin reuptake inhibitors (11 patients), valproic acid (2 patients) as well as carbamazepine and amitriptyline, each with one patient.

Diuretic use was also a common cause since it was at least one of the reasons contributing to hyponatraemia in 108 (27%) of the patients, and 100 of these patients were on hydrochlorothiazide or indapamide. Excessive alcohol intake, beer potomania or type of alcohol abuse, was the reason for hyponatraemia in 24% of the cases. Hypervolaemia explained hyponatraemia in 55 (14%) patients. A hepatic aetiology for hypervolaemia was more prevalent in the younger age quartiles, whereas cardiac insufficiency was more common in older patients.

Hyperglycaemia was the reason for low sodium concentration in 6 patients. In all cases, blood glucose was over 30 mmol/l, and 4 patients also had ketoacidosis. In addition, three patients’ serum was hyperosmolal due to severe dehydration, but no other hyperosmolal states were noted in this study.

The causes of hyponatraemia varied by age (Table 3). Older age increased the odds for severe hyponatraemia due to SIAD (OR for year increase 1.054, 95% CI 1.034–1.074, *p* < 0.001) and more specifically, subgroup of SIAD provoked by pain, nausea or vomiting (OR for year increase 1.036, 95% CI 1.010–1.062, *p* = 0.006) and SIAD with unspecified mechanism (OR for year increase 1.056, 95% CI 1.026–1.088, *p* < 0.001) were age-dependent. In addition, the proportion of patients with diuretic use as an aetiology of hyponatremia increased by age (OR for year increase 1.044, 95% CI 1.024–1.064, *p* < 0.001). Older patients were also more susceptible to develop severe hyponatremia after changes in medication (OR for year increase 1.029, 95% CI 1.010–1.048, *p* = 0.002) (Table 1). Cardiac hypervolemia was more frequent in older patients (OR for year increase 1.047, 95% CI 1.009–1.089, *p* = 0.016), whereas odds for hepatic hypervolemia decreased by age (OR for year increase 0.932, 95% CI 0.905–0.960, *p* < 0.001). Excessive alcohol use (OR for year increase 0.968, 95% CI 0.949–0.986, *p* < 0.001) was a more common cause in younger patients.

### Management of hyponatraemia

Seven different first-line treatments were initiated in the ED. Saline infusion was the most common choice, initiated for 61% of patients, followed by fluid restriction (18%), hypertonic saline bolus (6%), hypotonic fluid infusion (6%), other crystalloid infusion (5%). Hydrocortisone or antidiuretic hormone analogue was administered to one patient each. On average, patients received 2.6 different therapy modalities. Infusions of isotonic or hypotonic fluids and fluid restriction were widely used to control the rate of P-Na change. Antidiuretic hormone analogue was administered to 15% of patients, mostly in the ICU or HDU, to decelerate a too rapid increase in P-Na. Oral urea was initiated for SIAD patients who were resistant to a fluid restriction (*n* = 12, 3%).

On average, sodium was analysed 17 times (median 11, IQR 8–26) during the first 48 h, ranging from one to up to 50 measurements. The estimated sodium concentration at 24, 48 and 72 h after admission was calculated using the two closest analyses, one before and one after the time point, assuming that the change between them had occurred linearly. Data for calculation were available for 385, 375 and 363 patients at the 24-, 48- and 72-h time points, respectively.

During the first 24 h of hospitalization, the mean sodium correction rate was 6.6 mmol/l. Overall, 104 (27%) and 49 (13%) patients had a correction rate of > 8 mmol/l and > 10 mmol/l, respectively, at 24 h. During the second and third 24 h of hospitalization, the sodium correction rate was 4.9 mmol/l/24 h and 3.8 mmol/l/24 h, respectively (Table 4).

**Table 4:** Correction rates

		Median	(Range, IQR)	Mean	(SD)	No. of patients
Plasma sodium (mmol/l)						
Minimum		112	(98–115, 109–114)	111	(3.9)	394
24-h change at 24 h*		6.3	(-4 to 31, 4.0–8.9)	6.6	(4.1)	385
24-h change at 48 h*		5.0	(-7 to 15, 2.7–7.0)	4.9	(3.4)	375
24-h change at 72 h*		3.6	(-4 to 13, 1.9–5.7)	3.8	(2.9)	363
Maximum any 24-h change*		9.9	(0–32, 8–12)	10.2	(3.7)	
No. of analyses during the first 48 h		11	(1–50, 8–26)	17	(12.5)	

*An estimation using linear interpolation between sodium values prior to and following the timepoint

For every sodium analysis, an estimate of concentration change was calculated 24 h before and after the data point. The mean maximum change in P-Na during any 24-h period of the 72 h of hospitalization was 10.2 mmol/l, but there was a large variation in correction rates between patients.

### Mortality

The in-hospital mortality rate was 7.7% (28 of 363 individuals died), and the 30-day mortality rate was 10%.

Sixty-seven (18%) patients died within one year after the first admission to the ED due to hyponatraemia. The mean age of the deceased patients during their first ED admission was 68 years, the same as that of the whole study population.

Data on the cause of death were available for 61 patients (Table 5). Neurological causes of death included intracerebral haemorrhage (*n* = 3), stroke (*n* = 2) and status epilepticus (*n* = 1). In 22 (33%) patients, the disease leading to death was not diagnosed until after the first episode of severe hyponatraemia.

**Table 5:** Mortality rate and causes of death

1-year mortality	67	(18%)*
Malignancy	25	(37%)
Small cell lung cancer	12	(18%)
Liver disease	14	(21%)
Neurological	6	(9%)
Pneumonia	6	(9%)
Other	10	(15%)
Not known	6	(9%)

*Percentage of all patients, *n* = 363

### Osmotic demyelination syndrome

A brain MRI scan was performed on 39 (11%) patients during or after hospitalization due to hyponatraemia. The scans revealed 5 patients (1.4% of all individual patients in the study) with ODS. The detailed information on these patients is presented in Table 6. All of them used excessive amounts of alcohol, and 4 had simultaneous hypokalaemia. Thiamine substitution was administered to all patients with ODS during hospitalization. Charlson comorbidity index [13] ranged from 0 to 2 points, and none of patients had hepatic insufficiency. Phosphate concentration or nutritional status of the patients with ODS were not recorded.

**Table 6:** Patients diagnosed with osmotic demyelinisation syndrome

No	Age (years)	Sex	Alcohol abuse	Diuretic	Total no. of drugs	First inpatient location	P-K on admission (mmol/l)	P-Na (mmol/l)					No. of analyses during first 48 h
								On admission	At 24 h*	At 48 h*	At 72 h*	Maximum any 24-h change*	
1	49	Female	Yes		6	ICU	4.0	112	120	130	141	14	39
2	52	Male	Yes	FRS, SPL	4	ICU	2.8	104	115	122	131	14	47
3	55	Male	Yes	HCTZ	9	ICU	2.6	108	115	125	127	12	28
4	40	Female	Yes		0	ICU	2.5	108	114	118	127	11	29
5	53	Male	Yes	HCTZ	3	HDU	3.0	107	117	121	124	11	28

*FRS* furosemide, *HCTZ* hydrochlorothiazide, *HDU* internal medicine high-dependency unit, *ICU* intensive care unit, *SPL* spironolactone

^*^An estimation using linear interpolation between sodium values prior to and following the timepoint

P-Na was intensively monitored using arterial line sampling and a point-of-care blood gas analyser, and the mean interval between sodium analyses was less than 2 h in all patients during the first 48 h of care. The maximal sodium increase at any 24-h time point was from 11 to 14 mmol/l. However, when looking only at the exact 24-h, 48-h and 72-h time points after admission, the maximum increase rate was somewhat lower.

In this study, there were 145 patients whose maximum any 24-h sodium correction rate exceeded 10 mmol/l without causing ODS afterwards. Thus, ODS was observed in 3.3% of those at risk. The patients who did develop ODS were younger (mean age 50 vs 65 years), used more likely excessive amounts of alcohol (100% vs 39%) and were more often hypokalaemic upon admission (80% vs 19%) than those who did not develop ODS despite sodium rate overcorrection.

## Discussion

This study describes 394 ED visits by patients with severe hyponatraemia and is one of the largest case series ever reported. A literature search revealed one previous report in which the inclusion criteria were identical to the present study, i.e. P-Na < 116 mmol/l in the ED [14]. In addition, there are four studies that include a group of patients with slightly less severe hyponatraemia, P-Na < 120 or < 121 mmol/l [2, 4, 5, 12], which is still lower than the suggested cut-off for severe hyponatraemia in the European guideline [7]. According to pooled data from earlier reports comprising 1,907 patients, the mean age of patients with severe hyponatraemia was 67 years and there was a female predominance of 1.3:1. These findings well correspond to those of the present study (Table 1).

According to previous reports, patients with severe hyponatraemia present with non-specific symptoms [4, 5]. In the present study, 36% of the patients with severe hyponatraemia also reported a diffuse complaint of declined general performance. Although severe hyponatraemia is a potentially life-threatening electrolyte disorder, relying on measurements of vital signs may erroneously indicate a non-urgent situation. The NEWS is used to predict the eventual need for intensive care. In a study of unselected ED patients in our hospital, the median NEWS of patients admitted to the ICU was 7 [15], whereas in patients admitted to the ICU due to hyponatraemia, the median NEWS was 3. This emphasizes the important fact that in the great majority of severely hyponatraemic patients, hyponatraemia itself is not an immediate health risk. Instead, the correction of severe hyponatraemia by the initiation of untargeted or targeted therapy poses a potential risk of a varying degree to every patient [7].

The aetiology of severe hyponatraemia was multifactorial in 44% of cases, and, interestingly, hyponatraemia appears equally multifactorial in all age quartiles. SIAD was the most common mechanism contributing to hyponatraemia in 34% of the cases, followed by diuretic use (27%) and alcohol abuse (24%).

The proportions of SIAD, diuretic use and cardiac hypervolaemia were higher in older patients, whereas alcohol abuse and hepatic hypervolaemia were a more common underlying causes for hyponatraemia in younger age quartiles (Table 3). This finding of a varying aetiology of hyponatraemia by age is in line with clinical observations but has not been reported previously. Furthermore, the susceptibility to adverse events related to changes in medication increased with age.

The proportion of different aetiologies of severe hyponatraemia has been reported previously by Olsson et al. [4]. Despite a different mode of reporting, some comparisons with the present study are feasible. The multifactorial aetiology and proportion of diuretic use were almost equal in both studies, but in the present study, SIAD, alcohol abuse and polydipsia seem to be more prevalent, whereas hypovolaemia was less frequent.

The in-hospital, 30-day and one-year mortality rates in this study were 8%, 10% and 18%, respectively. Previously, rates between 7 and 13% have been reported for in-hospital mortality [4, 5, 8, 16], 14% for 30-day mortality and 22% one-year-mortality [2], which are consistent with the present study. Causes of death were available for 61 of the 67 deceased patients, and this is presumably the first study in which the causes of death are extensively reported. After a follow-up of one year, malignancies, especially small-cell lung cancer, or end-stage liver disease were the cause of death in over half of the deceased patients. Interestingly, in one-third of the deceased patients, the diagnosis of the disease leading to death was made only after the first episode of severe hyponatraemia.

The management of hyponatraemia was most often initiated with 0.9% saline or other isotonic infusion, or with fluid restriction. During hospitalization, variation in the management modalities increased due to more accurate diagnostics and the aim of maintaining the targeted correction rate.

The sodium correction rate was calculated at 24-h, at 48-h and 72-h time points using a linear interpolation between sodium values prior to and following the time point, a formula first described by George et al. [12]. In addition, the maximum 24-h correction rate at any time point during the first 72 h of hospitalization was calculated using the same interpolation method. The sodium correction rate is considered important since overcorrection is associated with the risk of developing ODS [17]. However, there is no standard for calculating the correction rate and no unequivocal definition for overcorrection. In the literature review by Woodfine and van Walraven [14], 9 different sodium correction rate formulae and 14 different criteria for overcorrection were found. The large variation in the calculation methods makes it difficult to interpret the association between the correction rate and ODS.

ODS was diagnosed by MRI in 5 patients, which represents 1.4% of all patients in this study (Table 6). The histories of all patients with ODS revealed alcohol abuse, and all but one were also hypokalaemic upon admission. In all patients, the sodium correction rate was intensively monitored. The maximum correction rate at the 24-h, 48-h, or 72-h time points was 11 mmol/l/24 h, and the maximum correction rate at any 24-h time point was from 11 to 14 mmol/l. When patients with ODS were compared to patients with similar overcorrection but no diagnosis of ODS, younger age, alcohol abuse and hypokalaemia upon admission were overrepresented.

Sodium overcorrection is not the only risk factor for ODS, which may develop even if the correction rate being within the recommended limits (≤ 10 mmol/l/day) [18]. Other proposed risk factors for ODS include hypokalaemia, hypophosphatemia, malnutrition, thiamine deficiency, alcoholism, and liver cirrhosis or transplantation [17–20]. In the present study, only alcohol abuse and hypokalaemia seemed to be contributing factors in developing ODS. There is still a need for solid data on safe sodium correction rates in various patient groups and comorbidities to guide clinicians who work with hyponatraemic patients.

### Limitations

This study is limited by its retrospective design and the method of obtaining information from patient records. The interpretation of factors causing hyponatraemia was performed by one experienced clinician only. In addition, the methodology did not allow blinding because it requires a thorough search through all patient records. Some important data were missing, which made it difficult to assess various aspects of hyponatraemia. This was especially the case in making the decision of the eventual aetiology of hyponatraemia, therefore these results should be interpreted with caution. However, the best effort to collect all relevant pieces of information was made to draw correct conclusions from the fate of each patient.

## Conclusions

In this series of 394 severely hyponatraemic (P-Na < 116 mmol/l) patients presenting at the ED, half of the patients had neurological symptoms, but non-specific complaints were also frequently reported. The aetiology of hyponatraemia was often multifactorial and varied by age. The value of vital signs, such as NEWS, in predicting the eventual need for intensive care was compromised in severely hyponatraemic patients. One-year mortality was 18%, and the main causes of death were malignancies, with small-cell lung cancer in particular, and end-stage liver disease. ODS was diagnosed in 5 (1.4%) patients, all of whom used excessive amounts of alcohol, and 4 out of 5 were also hypokalaemic upon admission.

## Data Availability

Data supporting this study cannot be made available due to sensitive and private information of individual patients.

## Abbreviations

CI: Confidence interval
ED: Emergency department
FRS: Furosemide
GCS: Glasgow coma scale
HCTZ: Hydrochlorothiazide
HDU: High-dependency unit
ICU: Intensive care unit
IQR: Inter-quartile range
MRI: Magnetic resonance imaging
NEWS: National early warning score
ODS: Osmotic demyelination syndrome
OR: Odds ratio
P-Na: Sodium concentration in plasma
PPI: Proton pump inhibitor
SD: Standard deviation
SIAD: Syndrome of inappropriate antidiuresis
SCLC: Small-cell lung cancer
SPL: Spironolactone
SSRI: Selective serotonin uptake inhibitor
